# *Schistosoma mansoni*–Induced Oxidative Stress Triggers Hepatocellular Proliferation

**DOI:** 10.1016/j.jcmgh.2023.08.014

**Published:** 2023-09-09

**Authors:** Verena von Bülow, Maryam Schneider, Dorothee Dreizler, Lena Russ, Anne Baier, Nicola Buss, Jakob Lichtenberger, Lukas Härle, Heike Müller, Annette Tschuschner, Gabriele Schramm, Jörn Pons-Kühnemann, Christoph G. Grevelding, Elke Roeb, Martin Roderfeld

**Affiliations:** 1 Department of Gastroenterology, Justus Liebig University Giessen, Giessen, Germany; 2 Early Life Origin of Chronic Lung Diseases, Priority Research Area Chronic Lung Diseases, Research Center Borstel, Borstel, Germany; 3 Institute of Medical Informatics, Justus Liebig University Giessen, Giessen, Germany; 4 Institute of Parasitology, Biomedizinisches Forschungszentrum Seltersberg, Justus Liebig University Giessen, Giessen, Germany

**Keywords:** Parasite, Cell Cycle, Replication Licensing, DNA Stress Response

## Abstract

**Background & Aims:**

Schistosomiasis is one of the most prominent parasite-induced infectious diseases, affecting more than 250 million people. *Schistosoma mansoni* causes metabolic exhaustion and a strong redox imbalance in the liver, causing parenchymal damage, and may predispose for cancer. We investigated whether oxidative stress provokes hepatocellular proliferation upon *S. mansoni* infection.

**Methods:**

The cell cycle, replication stress response, and proliferation were analyzed on transcriptional and protein levels in the livers of *S. mansoni*–infected hamsters and by mechanistic gain- and loss-of-function experiments in human hepatoma cells. Major results were validated in human biopsy specimens of *S. mansoni*–infected patients.

**Results:**

*S. mansoni* infection induced licensing factors of DNA replication and cell-cycle checkpoint cyclins in parallel with a DNA damage response in hamster hepatocytes. Moreover, even unisexual infection without egg effects, as a reflection of a chronic inflammatory process, resulted in a moderate activation of several cell-cycle markers. *S. mansoni* soluble egg antigens induced proliferation of human hepatoma cells that could be abolished by reduced glutathione.

**Conclusions:**

Our data suggest that hepatocellular proliferation is triggered by *S. mansoni* egg-induced oxidative stress.

## Graphical abstract

**Figure:**
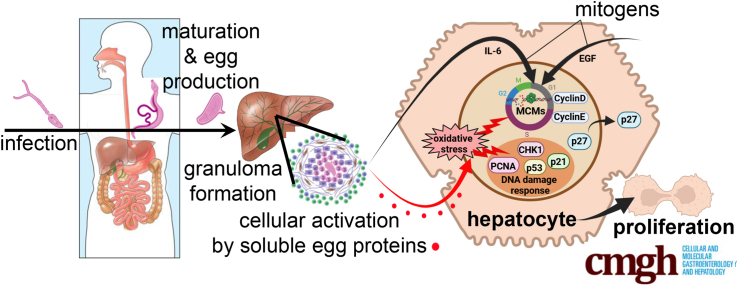

**Summary**

The control of hepatocellular proliferation by *Schistosoma mansoni* eggs is an essential aspect of the host–parasite interaction–associated liver damage. The results of the current study imply that *S. mansoni* soluble egg antigens trigger the hepatocellular cell cycle and promote proliferation.

Schistosomiasis is a parasitic disease caused by trematodes of the genus *Schistosoma*. With an estimated 251.4 million people affected in 2021, schistosomiasis is one of the most common parasitic diseases worldwide.^1^ Schistosomiasis-associated morbidity and mortality are responsible for serious medical and socioeconomic problems, especially in the global south. Recent epidemiologic studies described the occurrence of this neglected tropical disease also in Southern Europe, as evidenced by outbreaks of schistosomiasis in Corsica, France, and Almeria, Spain.^2^

During infection with *Schistosoma mansoni*, cercariae, one of the free-living stages of schistosomes that is released by intermediate host snails in fresh water, penetrate the skin of human beings as final hosts. After skin passage, cercariae enter the blood system as schistosomula, a juvenile form, before they develop into mature adult worms, which pair in the portal vein. As couples, *S. mansoni* worms migrate to the mesenteric complex of the intestine, where they produce up to 300 eggs per day for years.^3^ The eggs migrate from the mesenteric vessels into the intestinal lumen by inducing peri-ovular inflammation and are excreted by the host to continue the life cycle. However, up to 50% of the eggs fail to reach the gut lumen and travel with the blood flow to the liver, enter the tissue, and cause granulomatous hepatitis. In addition to acute fibrotic-inflammatory and granulomatous liver damage caused by the eggs, chronic liver damage is a major cause of hepatic morbidity and mortality worldwide.^4^ To date, praziquantel is the only effective drug in the treatment of schistosomiasis that effectively kills the adult worms, but not the eggs or the developing miracidial larvae. *S. mansoni* is a group 3 carcinogen for human beings, indicating insufficient evidence to determine its carcinogenicity.^5^ Nevertheless, *S. mansoni* soluble egg antigen (SEA), including the glycoprotein interleukin-4–inducing principle of *Schistosoma mansoni* egg/α-1 as a major component, activate oncogenic signaling and DNA damage in the liver and colon.^6^^,^^7^ *S. mansoni* eggs are even able to mobilize, incorporate, and store host lipids, thereby provoking hepatic exhaustion of neutral lipids and glycogen.^8^ The associated metabolic reprogramming causes oxidative stress–induced DNA damage in hepatocytes. High levels of reactive oxygen species (ROS) induce oncogenic signaling and even may promote malignancies by DNA damage.^9^ To maintain the stability of the genome it is mandatory that eukaryotic DNA replication is strictly limited to only once per cell cycle. This is ensured by several mechanisms that control the assembly of the pre-replication complex, a process known as replication licensing.^10^ In the early G1 phase, cell division cycle 6 (CDC6) is recruited to the origin through interaction with the origin recognition complex.^11^ Subsequently, CDC6 recruits chromatin licensing and DNA replication factor 1 to the origin recognition complex, building a platform for the loading of the minichromosome maintenance factor (MCM)2–7 DNA helicase complex onto chromatin in the late G1 phase.^12^ Thus, the origins are ready for replication initiation of the S phase. Inactivation of these licensing factors is crucial for preventing the re-initiation of DNA replication during the next cell-cycle steps. Aberrant expression and activation of MCMs are detected frequently in various premalignant lesions and malignancies, leading to genome instability and uncontrolled cell-cycle progression.^13^

The orderly progression of the 4 phases of the cell-cycle is ensured by checkpoint regulators. Abnormal functioning of checkpoints and the inability to recognize damaged DNA may play an important role in tumor initiation and progression by allowing cells to proceed through the cell-cycle with damaged or abnormal DNA. Human cyclin D1 has been shown to be the primary regulator and mandatory for inducing transcription and activation of proteins associated with passage through the G1 checkpoint and progression into the S phase.^14^ Overexpression of human cyclin D1 has been associated with disordered proliferation and reported in several primary human cancers, including hepatocellular carcinoma, supporting its role as an oncogene.^15^ Another important regulator of the G1/S checkpoint is p27^kinase^ ^inhibitor protein^ ^1^^(KIP^^1^^)^, which is an anti-oncogene for cell-cycle regulation, and thus functions as a negative regulator. Increased ROS levels and DNA damage activate a replication stress response that enables the cell to maintain the integrity of the genome. The DNA damage response activates cell-cycle checkpoint regulators such as checkpoint kinase (CHK), which delay cell-cycle progression to allow DNA repair.^16^

Whether *S. mansoni* egg-secreted proteins may trigger abnormal expression of cell-cycle checkpoint regulators in hepatocytes is not known to date.

The current study shows that infection with *S. mansoni* disrupts the cell-cycle control in hepatocytes of infected hamsters. Furthermore, SEA induces cell proliferation of cultured human hepatoma cells that was abolished by additional treatment with reduced glutathione (GSH).

## Results

### *S. mansoni* Infection Modulated the Expression of Hepatic Replication Licensing Proteins

**Figure 1:**
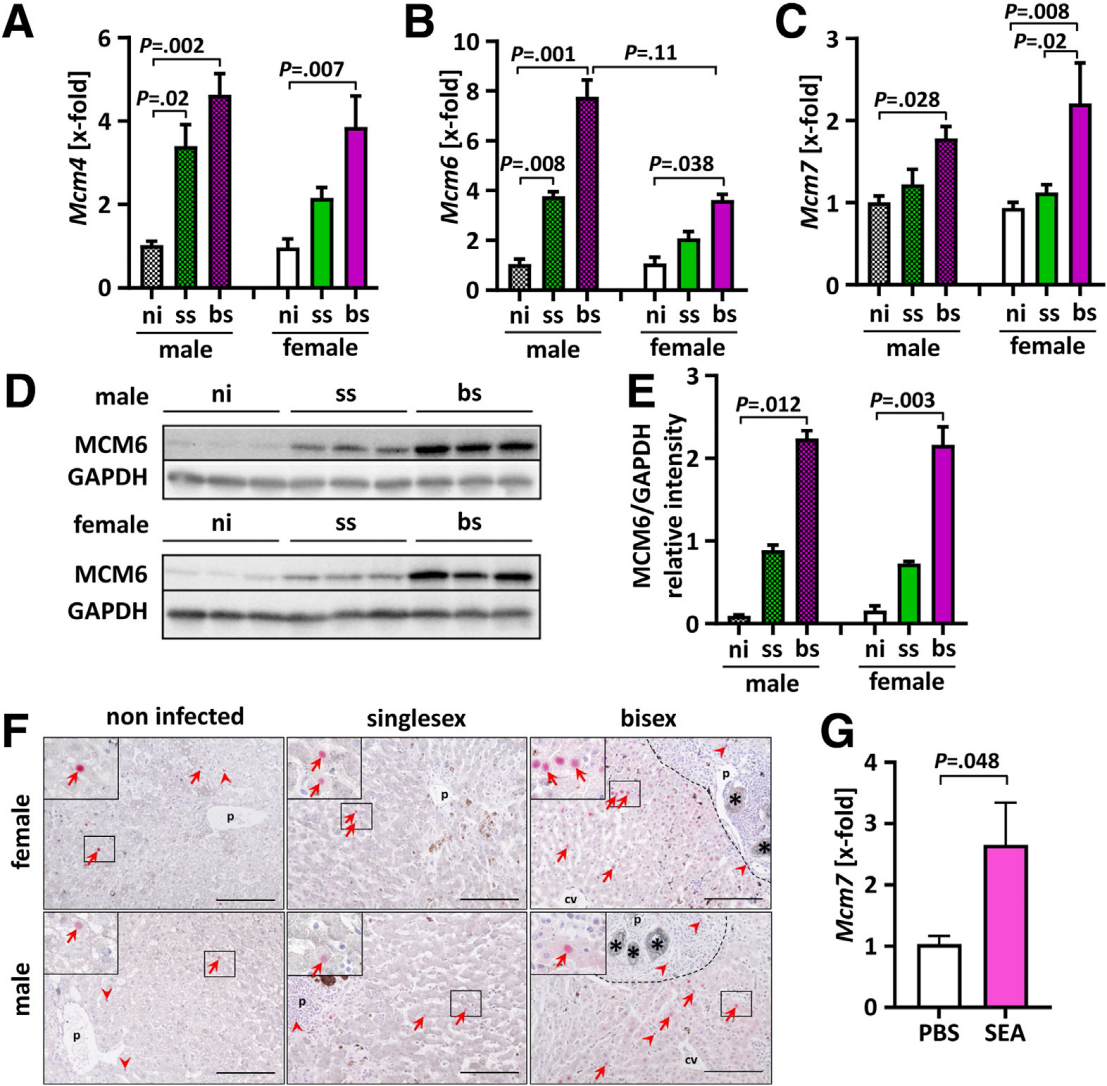
***S. mansoni* infection caused aberrant expression of hepatic MCM proteins.** (*A–C*) Hepatic mRNA levels of minichromosome maintenance protein (*Mcm*) genes 4, 6, and 7 were increased in bs-infected hamsters of both sexes (n = 3–5). (*D* and *E*) Western blot analysis and densitometric analysis showed the induction of MCM6 in livers of bs-infected hamsters (n = 3). A representative Western blot is shown. (*A–E*) Data were normalized to the control (ni) group, and the Kruskal–Wallis test was performed to assess group differences. (*F*) MCM6 (red)/fibrinogen (grey) co-immunostaining of histologic liver sections showed low expression levels of MCM6 in ni animals and ss-infected hamsters, as well as the increased nuclear accumulation of MCM6 in perigranulomatous hepatocytes (*red arrows*) and to a lesser extent in leukocytes (*red arrowheads*) of bs-infected hamsters. The acute-phase protein fibrinogen is synthesized in hepatocytes. Here, it stained the cytoplasm of hepatocytes light grey. This allows differentiation between parenchymal MCM6 activation (*red arrows*) in hepatocytes and MCM6 activation in nonparenchyma cells (*red arrowheads*). Representative hepatic co-immunostainings of female and male noninfected, ss-infected, and bs-infected hamsters are shown. Original magnification: 200×. *Scale bar*: 100 μm. *Black dotted line* indicates a granuloma. ∗ *S. mansoni* egg. (*G*) The levels of *Mcm7* mRNA increased in HepG2 cells treated with *S. mansoni* SEA. Data were normalized to the control (phosphate-buffered saline [PBS]) group. These experiments were performed at least 3 times independently. Levels of significance are indicated in the figure (Student *t* test). cv, central vein; GAPDH, glyceraldehyde-3-phosphate dehydrogenase; p, portal tract.

MCM proteins play essential roles in the initiation of DNA synthesis and are indispensable for licensing of DNA replication to safeguard one replication round per cell cycle.^17^ Consequently, dysregulated MCMs lead to cell-cycle disturbance and eventually to tumor initiation, progression, and chemical resistance.^13^ Previous studies from our laboratories provided evidence for an association of the presence of *S. mansoni* eggs in liver tissue as one prerequisite for the possibility of the onset of malignant transformation.^8^ We now investigated whether *S. mansoni* eggs may influence expression of hepatic MCM proteins (Figure 1). Analysis of messenger RNA (mRNA) expression levels of *Mcm 4*, *6*, and *7*, as well as Western blot analysis of hepatic MCM6 protein, consistently showed that *S. mansoni* infection induced a strong up-regulation of hepatic MCMs without significant gender differences (Figure 1*A–E*). As expected, in bisex (bs)-infected hamsters (final host infections with both schistosome genders), in which massive egg production occurs as a result of the presence of couples, we observed a significant up-regulation of the transcript levels of *Mcm 4*, *6*, and *7*. Remarkably, single-sex (ss) infections (final host infections with 1 schistosome gender only), in which no eggs were detected, also caused some up-regulation of gene expression, which was significant for *Mcm 4* and *Mcm 6* in male hamsters (Figure 1*A* and *B*).

The additional Western blot result indicated a significant up-regulation of MCM6 in bs-infected female and male hamsters (Figure 1*D* and *E*). Furthermore, nuclear staining of MCM6 was enhanced in perigranulomatous hepatocytes (Figure 1*F*, red arrows), especially in bs-infected hamsters. Among the MCMs, MCM7 is a major subunit of the putative heteromeric MCM helicase complex and has been reported to be involved in tumor development and progression.^18^ Therefore, we investigated whether *Mcm7* mRNA expression could be stimulated by *S. mansoni* egg antigens in human hepatoma cells. Figure 1*G* shows that SEA caused an increase of the *Mcm7* mRNA level in the human hepatoblastoma cell line HepG2. This suggests that egg-secreted factors may enhance hepatocellular replication licensing factors.

### *S. mansoni* Infections Triggered the Replication Stress Response

**Figure 2:**
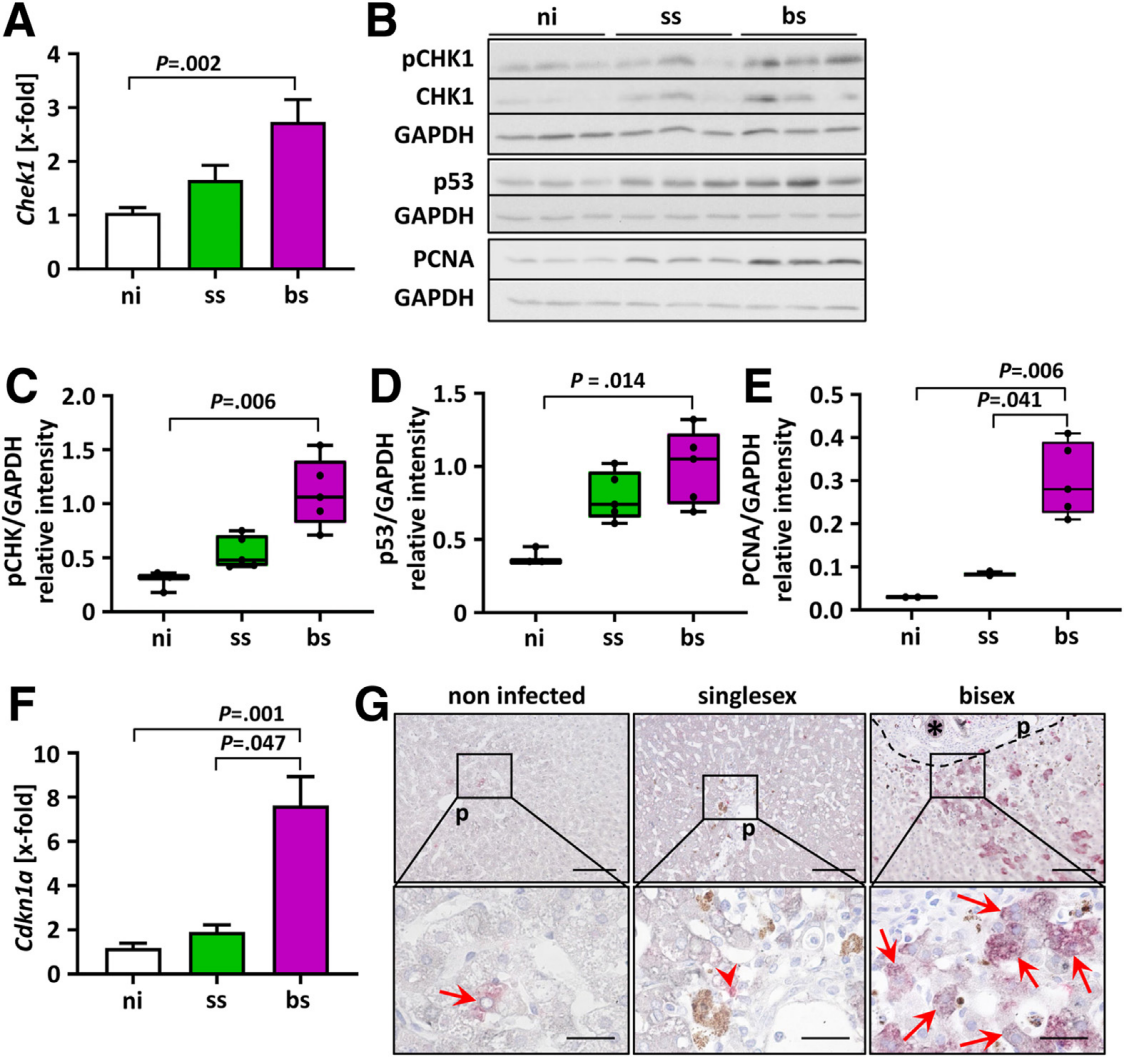
***S. mansoni* infection triggered the replication stress response.** (*A*) Gene expression (mRNA level) of checkpoint kinase 1 (*Chek1*) was up-regulated in livers of bs-infected hamsters (n = 5–8). Data were normalized to the control group, and the Kruskal–Wallis test was performed to assess group differences. (*B* and *C*) Phosphorylation of CHK1 at Ser345 revealed checkpoint activation in livers of bs-infected hamsters as determined by Western blot analysis and densitometric analysis. (*B* and *D*) The tumor-suppressor protein p53 was up-regulated in livers of bs-infected hamsters as determined by Western blot analysis and densitometric analysis. (*B* and *E*) PCNA was increased at the protein level in livers of bs-infected hamsters. (*F*) The mRNA expression levels of cyclin-dependent kinase inhibitor p21^SDI1^ (*Cdkn1a*), essential for DNA damage response, were increased in livers of bs-infected hamsters (n = 5–8). (*G*) Representative co-immunostainings for p21^SDI1^ (red)/fibrinogen (grey) in the livers of female hamsters, which were noninfected (control), ss-infected, and bs-infected. The acute-phase protein fibrinogen is synthesized in hepatocytes and here stained the cytoplasm of hepatocytes light grey. This allows identification of parenchymal p21 expression (*arrows*) in hepatocytes. *Arrows* indicate p21 staining in hepatocellular cytoplasm, while *arrowheads* point out p21-stained nonparenchyma cells. Of note, p21^SDI1^ protein was detected mainly in the cytoplasm of hepatocytes in bs-infected hamsters. Original magnification: 200× and 1000×. *Scale bars*: 100 and 25 μm. ∗Eggs. *Dashed line* indicates the granuloma border. Representative Western blots are depicted. Data were normalized to the control group, and the Kruskal–Wallis test was performed to assess group differences. Levels of significance are indicated in the figure. GAPDH, glyceraldehyde-3-phosphate dehydrogenase; ni, noninfected; p, portal tract; pCHK1, phosphorylated checkpoint kinase 1.

Next, we investigated whether *S. mansoni* infection leads to the induction of a hepatic DNA damage response. Because no significant gender-specific differences in the regulation of the licensing factors were found, we focused on female hamster livers in the following experiments. A crucial component of the DNA damage response is the checkpoint kinase CHK1, which activates DNA damage checkpoint and cell-cycle arrest.^19^ Hepatic expression of the *Chek1* gene was up-regulated by *S. mansoni* infection in livers of bs-infected hamsters only (Figure 2*A*).

Subsequently, we showed that *S. mansoni* infection induced the activation of CHK1 as shown by phosphorylation at Ser345 (Figure 2*B* and *C*). The expression level of the tumor-suppressor p53 was increased significantly in livers of bs-infected hamsters (Figure 2*B* and *D*). In addition, the expression level of hepatic DNA polymerase processivity factor proliferating cell nuclear antigen (PCNA), which serves as the master coordinator of DNA replication and repair, was enhanced upon *S. mansoni* infection, most significantly upon bs-infection (Figure 2*B* and *E*). Subsequently, the gene (*Cdkn1a*) expression level of the cell-cycle inhibitor p21^senescence DNA synthesis inhibitor 1(SDI1^^)^, the transcriptional target of p53,^20^ was up-regulated significantly at the mRNA level in *S. mansoni*–infected hamsters even compared with ss-infection (Figure 2*F*). However, co-immunohistochemistry staining showed p21^SDI1^- (red)/fibrinogen (grey)-positive hepatocytes mostly in livers of bs-infected hamsters. Staining usually was located in the cytoplasm (Figure 2*G*).

### *S. mansoni* Infection Induced Hepatic Cell-Cycle Checkpoint Regulators

**Figure 3:**
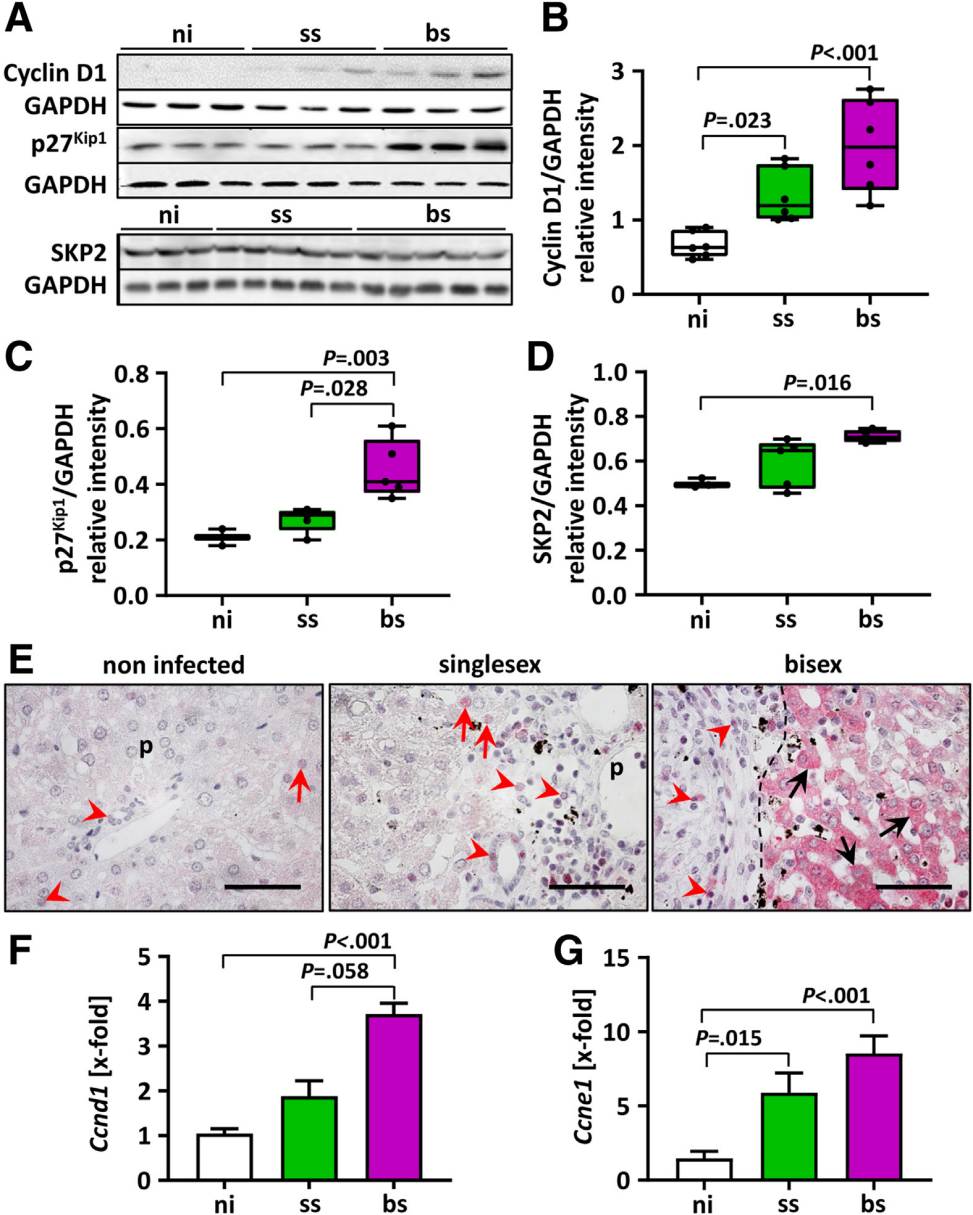
***S. mansoni* infection provoked up-regulation of G1/S checkpoint regulators.** (*A* and *B*) Hepatic cyclin D1 was strongly up-regulated in livers of bs-infected hamsters as shown by Western blot analysis (n = 6). (*A* and *C*) *S. mansoni* bs-infection caused an increase of the hepatic cyclin-dependent kinase inhibitor p27^KIP1^ compared with ni- and ss-infected hamsters (n = 3–5). (*A* and *C*) As shown by Western blot, hepatic SKP2 protein levels were up-regulated in bs-infected hamsters compared with ss-infected and noninfected controls (n = 3–5). (*A*) Representative Western blots are shown. (*B–D*) Data were normalized to the control group, and the Kruskal–Wallis test was performed to assess group differences. (*E*) Representative immunostaining for p27^KIP1^ in the livers of female hamsters, noninfected, ss-infected, and bs-infected. *Red arrows* indicate p27^KIP1^-positive hepatocellular nuclei, while *arrowheads* point out p27^KIP1^-stained nuclei of leukocytes. Please note p27^KIP1^ products in the cytoplasm of hepatocytes of bs-infected hamsters (*black arrows*). Original magnification, 1000×. *Scale bars*: 50 μm. ∗Eggs. *Dashed line* indicates the granuloma border. (*F* and *G*) Hepatic *cyclin D1* and (C) *cyclin E1* gene (*Ccbnd1*, *Ccne1*) expression was increased significantly in bs-infected hamsters (n = 5–8). GAPDH, glyceraldehyde-3-phosphate dehydrogenase; ni, noninfected; p, portal tract; SKP2, S phase kinase-associated protein 2.

Cyclin D1 is a key player that controls the transition from G1 to S phase, a key event in cell-cycle progression.^14^ To analyze the influence of *S. mansoni* infection on the cell cycle in liver cells, we investigated cyclin D1 expression levels in liver samples. Cyclin D1 protein levels were increased strongly by *S. mansoni* infection (Figure 3*A* and *B*). Cyclin D1 also was increased in livers of ss-infected hamsters.

Cell-cycle progression is negatively controlled by members of the KIP family. One family member, the cyclin-dependent kinase (CDK) inhibitor and tumor-suppressor p27^Kip1^, is critical for controlling the transition of G1/S phase to inhibit replication during DNA repair. Western blot analysis revealed augmented hepatic p27^KIP^^1^ protein levels in bs-infected compared with noninfected and ss-infected hamsters (Figure 3*A* and *C*). In parallel, the expression level of the hepatic S-phase kinase-associated protein 2 was up-regulated significantly in livers of bs-infected hamsters (Figure 3*A* and *D*). p27^KIP1^ functions at the nuclear level by binding to and inhibiting cyclin/CDK complexes, while cytoplasmic displacement inhibits p27^KIP^^1^ activity.^21^ Indeed, positive immunostaining of p27^KIP^^1^ in livers of bs-infected hamsters was detected mainly in the cytoplasm of perigranulomatous hepatocytes compared with controls. This strongly suggests a displacement-associated inactivation of p27^KIP^^1^ (Figure 3*E*).

Another key regulator of cell-cycle progression, accumulating at late G1 and promoting S-phase entry, is cyclin E.^22^ Cyclin D1 gene (*Ccnd1*) expression was induced in bs-infected hamsters (Figure 3*F*), whereas cyclin E1 gene (*Ccne1*) expression increased significantly in liver lysates of bs-infected hamsters (Figure 3*G*). Increased transcript levels also were found in livers of ss-infected hamsters.

### Increased Proliferation of SEA-Stimulated Hepatoma Cells Is Abrogated by GSH

**Figure 4:**
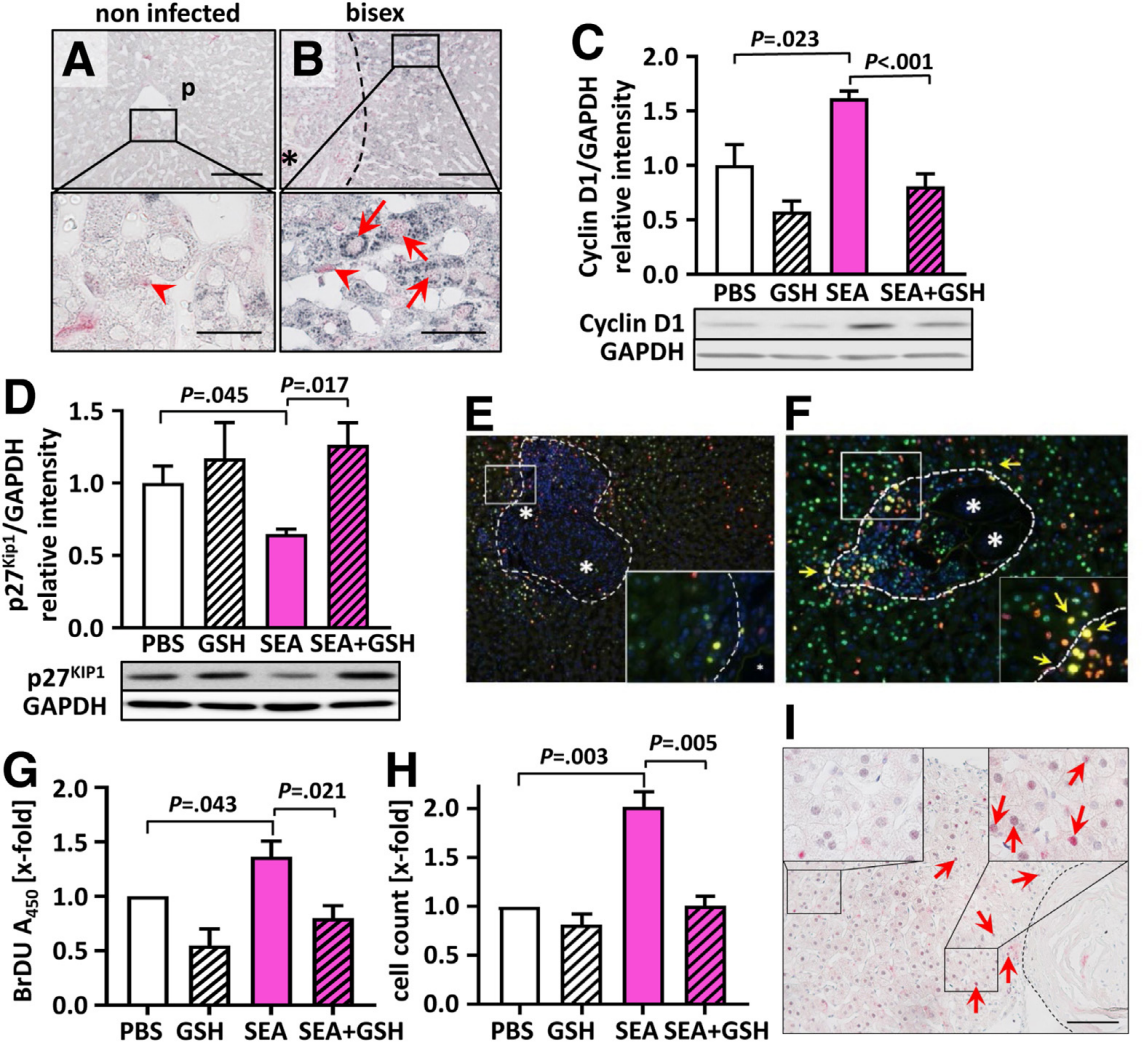
**Increased proliferation of SEA-stimulated hepatoma cells is abrogated by GSH.** (*A* and *B*) Representative co-immunostaining of SOD2 and MCM7 in the livers of female hamsters, which were (*A*) ni-infected (control) and (*B*) bs-infected. *Arrows* indicate MCM7 staining (red) in perigranulomatous hepatocellular nuclei with strong cytoplasmic SOD2 staining (grey), while *arrowheads* point out MCM7-stained nuclei of nonparenchyma cells. Please note the co-expression of cytoplasmic SOD2 and nuclear MCM7 in perigranulomatous hepatocytes. Original magnification, 200× and 1000×. *Scale bars*: 100 and 50 μm. ∗Eggs. *Dashed line* indicates the granuloma border. (*C*) SEA treatment induced the up-regulation of cyclin D1 protein levels in HepG2 cells and their down-regulation to control levels by the addition of the reduction equivalent GSH. Cell-culture experiments were performed 5 times independently. (*D*) SEA treatment stimulated down-regulation of the p27^KIP1^ protein level in HepG2 cells, and its up-regulation to control levels by the addition of the reduction equivalent GSH. Cell culture experiments were performed 3 times independently. (*C* and *D*) Representative Western blots are depicted. (*E* and *F*) Co-staining of the nuclei with Ki67 and the phosphorylated histone variant H2AX (γH2AX) showed proliferating hepatocytes with DNA damage in livers of *S mansoni* bs-infected hamsters. The 2 liver tissue sections were stained with double-staining multiplex immunohistochemistry. Proliferation of HepG2 cells increased by SEA treatment and remained at basal levels by the addition of reduction equivalents in the form of GSH as shown by (*G*) BrdU proliferation assay and (*H*) cell count. Data were normalized to the control (phosphate-buffered saline [PBS]) group. These experiments were performed at least (*G*) 4 and (*H*) 3 times independently. Levels of significance are indicated in the figure (Student *t* test). (*I*) Perigranulomatous hepatocellular cyclin D1 expression in a patient infected with *S. mansoni.* Human liver biopsy co-immunohistologically stained for cyclin D1 (red) and hepatocyte nuclear factor 4 alpha (HNF4α) (grey). Original magnification, 200×. *Scale bar*: 100 μm. *Dashed line* indicates a granuloma. *Box* is the area shown with higher resolution. *Red arrow* indicates hepatocellular nuclei positively stained for cyclin D1. GAPDH, glyceraldehyde-3-phosphate dehydrogenase; ni, noninfected; p, portal tract.

We next tested whether *S. mansoni* infection–induced oxidative stress triggered cell-cycle progression. To this end, we analyzed superoxide dismutase 2 (SOD2) and MCM7 expression by immunohistochemistry staining in liver tissue of *S. mansoni*–infected hamsters (Figure 4*A* and *B*).

Notably, both cytoplasmic SOD2 and nuclear MCM7 expression appeared markedly increased, especially in perigranulomatous hepatocytes near schistosome eggs. SOD2 also was detected in leukocytes of bs-infected livers, reflecting that oxidative stress is predominant in immune cells and parenchyma cells (Figure 4*B*). Figure 4*C* shows that SEA stimulation of HepG2 cells led to induction of cyclin D1 expression *in vitro*. This effect was reversed by the addition of the reducing agent *laevus* enantiomer of GSH (L-GSH). In parallel, SEA caused a down-regulation of p27^KIP1^ expression, which was normalized to control levels by L-GSH (Figure 4*D*). Furthermore, the phosphorylated histone variant H2AX (γH2AX), a marker for replication stress and repair of DNA double-strand breaks, and the marker of proliferation Kiel 67 (Ki67), were shown to colocalize within the same perigranulomatous nuclei of hepatocytes in bs-infected hamsters (Figure 4*E* and *F*). In line with these findings, we showed by bromodeoxyuridine (BrdU) and proliferation assays that SEA-induced proliferation was reversed *in vitro* by the ROS scavenger L-GSH (Figure 4*G* and *H*). Finally, immunohistochemistry analysis of liver tissue from a patient infected with *S. mansoni* also provided evidence of perigranulomatous hepatocellular nuclear cyclin D1 accumulation (Figure 4*I*, magnified in the upper right box).

### Induction of Paracrine Mitogens in the Liver of *S. mansoni*–Infected Hamsters

In the preceding sections, we have shown that proliferation of hepatoma cells was stimulated directly by proteins of the parasite. Because inflammatory cytokines in combination with growth factors can induce the cell cycle in hepatocytes, we studied the expression of interleukin 6 (IL-6) and epidermal growth factor (EGF) in livers of *S. mansoni*–infected hamsters.

**Figure 5:**
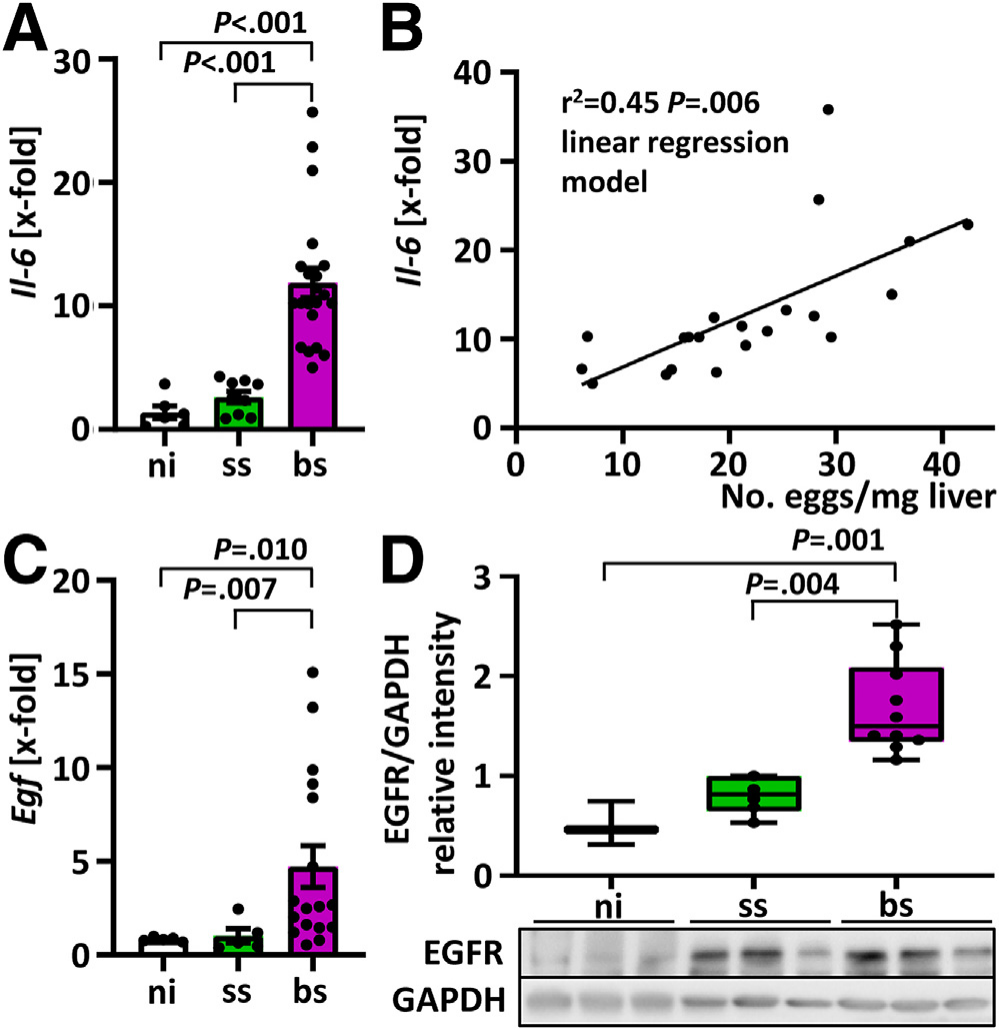
***S. mansoni* infection enhanced mitogen expression in hamster livers.** (*A*) Hepatic *Il-6* expression was increased significantly in bs-infected hamsters compared with ss-infected and ni-infected controls (n = 6–22). (*B*) Hepatic *Il-6* expression levels increased significantly with the hepatic egg burden in bs-infected hamsters. (*C*) Hepatic expression of *Egf* was increased significantly compared with ss-infected and ni-infected controls (n = 5–17). Hepatic EGFR protein levels were increased significantly by bs infection (n = 3–5). A representative Western blot is depicted. Data were normalized to the control group, and the Kruskal–Wallis test was performed to assess group differences. GAPDH, glyceraldehyde-3-phosphate dehydrogenase; ni, noninfected.

Expression levels of *Il6* were increased significantly in the bs-infected group (Figure 5*A*) and correlated positively with hepatic egg burden (Figure 5*B*). Moreover, we detected significantly increased *Egf* expression levels in livers of bs-infected animals (Figure 5*C*). Analyzing the stress sensor epidermal growth factor receptor (EGFR) also showed a strong up-regulation of expression in livers of *S. mansoni*–infected hamsters (Figure 5*D*).

## Discussion

In this study we have shown that an infection with *S. mansoni* leads to the induction of cell-cycle progression in hamster hepatocytes. Furthermore, *S. mansoni* egg antigen-induced proliferation of human hepatoma cells was normalized by the additional application of GSH. Furthermore, colocalization of cell-cycle progression markers together with markers for oxidative stress or DNA damage in perigranulomatous hepatocytes of *S. mansoni*–infected hamsters were presented. Finally, nuclear expression of cyclin D1 in liver samples of infected patients validated the results from model systems.

Cell-cycle progression in livers of *S. mansoni*–infected hamsters was characterized by increased expression of the replication licensing factors, the MCM proteins. Furthermore, cell culture experiments showed that even isolated egg proteins alone are sufficient to induce expression of MCM7 in human hepatoma cells without the involvement of the generally accepted paracrine-triggered mechanism by mitogens such as interleukin 6 and EGF. Improper control of replication initiation can be a source of replication stress.^23^ MCM proteins are mandatory for DNA licensing to permit the DNA to replicate only once per cell cycle. Furthermore, several MCM proteins have been found to be associated closely with tumorigenesis.^24^ Among them, MCM7 is implicated in tumor development and progression, and is considered as a potential biomarker for a variety of human malignancies.^18^ In hepatocellular carcinoma, increased expression of MCM7 correlated significantly with intrahepatic metastasis and vascular invasion.^25^ Furthermore, MCM7 was shown to act as an inducer for hepatic cyclin D1 via activation of the mitogen-activated protein kinase signaling pathway including extracellular signal–regulated kinase (ERK), c-Jun-N-terminal kinase, and p38.^26^ Recently, we reported that *S. mansoni* SEA activates hepatocellular oncogenic signaling including ERK and c-Jun-N-terminal kinase/c-Jun-pathways by oxidative stress.^6^^,^^8^ We discovered infection-induced hepatic expression of EGF and its receptor EGFR. EGFR-mediated activation of the ERK1/2 signal cascade also could contribute to cyclin D1 induction.^27^ Nevertheless, it also has been shown that cyclin D1 expression is sufficient to promote cell-cycle progression in the absence of mitogens in hepatocytes.^28^

We observed increased expression of p27^KIP1^ in *S. mansoni* hamster livers. However, the subcellular localization occurred mainly in the cytoplasm of perigranulomatous hepatocytes. Cytoplasmic p27^KIP1^ localization indicates its inactivation. In addition to the canonical function of p27^KIP1^ as a CDK inhibitor, which controls G1/S phase to inhibit cell proliferation, many studies have described the role of noncanonical p27^KIP1^ functions independent of CDK/cyclin regulation.^29^ A key feature of the noncanonical activity of p27^KIP1^ is its cytoplasmic localization, which has been shown as involvement in cellular processes such as cytoskeletal dynamics, cell migration, and metastasis.^30^ Interestingly, the expression of p27^KIP1^ was reduced by stimulation of human hepatoma cells with SEA.

Increased oxidative stress during an infection with *S. mansoni* is attributed not only to the inflammatory response of granulomatous immune cells, but also to the metabolic exhaustion caused by *S. mansoni* eggs in the liver.^8^ Thus, during infection, hepatocytes constantly are confronted with oxidative stress, which can induce DNA damage. Replication stress response allows cells to preserve the integrity of the genome by transiently slowing down or stalling replication forks.^16^ We detected both increased expression and increased activation of CHK1 by phosphorylation at Ser345. In response to DNA damage, CHK1 is phosphorylated and activated by ataxia telangiectasia and Rad3-related protein kinase at Ser345 to facilitate DNA repair.^31^

p53 activity is stimulated by DNA damage via activation of the ataxia-telangiectasia mutated/ataxia telangiectasia and Rad3-related and CHK1/CHK2 protein kinases.^32^ As a result, p53 induces cell-cycle arrest, allows DNA repair, and thus protects genome stability. Other stimuli, including oxidative stress and nutrient deprivation or the activation of oncogenes, can induce the expression of p53-dependent genes, facilitating the consequences of stress.^33^ Consequently, in response to moderate stress, p53 stimulates the expression of prosurvival genes that protect cells from damage, while severe stress stimulates cell death or senescence pathways. In addition, activated p53 induces the expression of p21^SDI1^,^34^ a cell-cycle regulatory protein essential for DNA damage response.^35^ p21^SDI1^ inhibits the cyclin D/CDK4 and cyclin E/CDK2 complexes, blocking the phosphorylation of protein substrates that are essential for the onset of the S phase.^36^ PCNA is central for both DNA replication and repair. PCNA activity is inhibited by p21^SDI1^.^37^ However, overexpression of p53 could disrupt this signaling pathway and trigger PCNA activity, thereby promoting cancer cell proliferation.^38^ In conclusion, the hepatic dysregulation of these factors in *S. mansoni*–infected hamsters suggests proliferative stress.

Our study provides evidence that cell proliferation of hepatoma cells is triggered by *S. mansoni* egg antigen–induced oxidative stress. The induction of cell-cycle markers was shown in bs-infected hamsters and even for some distinct factors in livers of ss-infected hamsters. Replication stress response genes were induced in parallel with the activation of the cell cycle. In summary, our data allow us to conclude that infection with *S. mansoni* leads to hepatocyte proliferation.

## Methods

### Human Material

Pseudonymized human liver samples were provided by the tissue bank of the German Center for Infection Research after approval by the ethics committee (project ID: 0064). According to the ethics vote, informed consent was not required for our retrospective analyses of archived tissues.

### Animal Experimentation

*Biomphalaria glabrata* snails served as intermediate hosts and Syrian hamsters (*Mesocricetus auratus*) were final hosts for maintaining the life cycle of a Liberian strain of *S. mansoni*. Worm populations (bs and ss) were generated by polymiracidial and monomiracidial intermediate host infections, respectively. Bs infections (n = 22) were performed at the age of 8 weeks and were maintained for 46 days. Ss infections (n = 7) were maintained for 67 days to ensure complete maturation of the worms; females need longer to grow and develop in the absence of male partners. Untreated hamsters (n = 6) were used as supercontrols. All animal experiments were performed in accordance with the European Convention for the Protection of Vertebrate Animals used for experimental and other scientific purposes (ETS no. 123; revised Appendix A) and were approved by the Regional Council Giessen (V54-19 c 20/15c GI 18/10 Nr. A26/2018).

### Isolation of Soluble Egg Antigens

*S. mansoni* eggs were obtained from livers of bs-infected hamsters on day 46 after infection, and SEA was isolated as described previously.^39^

### Quantification of *S mansoni* Eggs

Liver tissue (100 mg) of bs-infected hamsters (n = 22) was digested in 5% potassium hydroxide at 37°C for 16 hours.^40^ Subsequently, eggs were counted independently by 2 persons 3 times per sample. The number of eggs was calculated per milligram of liver tissue.

### Cell Culture Experiments

HepG2 cells (stock ordered in 2019, CLS #330198, expanded and stored as cryostocks for consistent quality in culture for up to 10 passages per cryostock) were stimulated with 15 μg/mL SEA and/or 10 mmol/L reduced L-glutathione (GSH) for the indicated time points.

### BrdU Assay and Cell Count

HepG2 cells were stimulated with SEA and/or GSH for 48 hours. BrdU was added 12 hours before the end of stimulation and cell proliferation was determined by the Cell Proliferation Enzyme-Linked Immunosorbent Assay Kit (Abcam) according to the manufacturer's protocol. HepG2 cells were harvested 48 hours after stimulation and cell count was performed in a Neubauer Counting Chamber (LO – Laboroptik GmbH).

### Quantitative Real-Time Polymerase Chain Reaction

mRNA isolation, transcription, quantitative real-time polymerase chain reaction, and data analysis were performed as described previously.^41^

### Western Blot Analysis

Western blot was performed as described previously.^42^

### Immunohistochemistry

Immunohistochemical detections were performed as described previously.^43^ Co-immunostaining was performed on formalin-fixed, paraffin-embedded, liver tissue. After heat-induced epitope retrieval, using citrate buffer, the slides were incubated with polyclonal rabbit antifibrinogen (27913; Abcam) in combination with polyclonal goat anti-MCM6 (9843; Santa Cruz) and polyclonal goat anti-p21 (397G; Santa Cruz). After incubation with enzyme-linked secondary antibodies, the development was performed with Vector SG (SK-4705; Vector Laboratories) and Permanent Red (ZUC001-125; Zytomed Systems).

### Statistical Analysis

The present study was exploratory. The study was performed with an existing number of cryopreserved organs that were not required for the maintenance of the parasite life cycle. Statistical analysis was performed with the Kruskal–Wallis test and the Student *t* test using GraphPad Prism version 5.3.1 (GraphPad Software, LLC, d.b.a Dotmatics). Because of the exploratory nature of the study, no further adjustment for *P* values was performed. Densitometrically assessed data from Western blots (hamster liver) are depicted as means ± 95% CIs.
